# Secular trend in dietary patterns of Iranian adults from 2006 to 2017: Tehran lipid and glucose study

**DOI:** 10.1186/s12937-020-00624-x

**Published:** 2020-10-03

**Authors:** Maryam Aghayan, Golaleh Asghari, Emad Yuzbashian, Maryam Mahdavi, Parvin Mirmiran, Fereidoun Azizi

**Affiliations:** 1 Nutrition and Endocrine Research Center, Research Institute for Endocrine Sciences, Shahid Beheshti University of Medical Sciences, P.O. Box: 19395-4763, Tehran, Iran; 2 Department of Clinical Nutrition and Dietetics, Faculty of Nutrition Sciences and Food Technology, National Nutrition and Food Technology Research Institute, Shahid Beheshti University of Medical Sciences, Tehran, Iran; 3 Obesity Research Center, Research Institute for Endocrine Sciences, Shahid Beheshti University of Medical Sciences, Tehran, Iran; 4 Endocrine Research Center, Research Institute for Endocrine Sciences, Shahid Beheshti University of Medical Sciences, Tehran, Iran

**Keywords:** Dietary patterns, Secular trend, Nutrition transition, Factor analysis, Adults, TLGS

## Abstract

**Background:**

Based on data regarding nutrition transition in the Middle East and North Africa, this study aim to investigate the general structure and secular trend of dietary patterns reported from the Tehran Lipid and Glucose Study (TLGS) and adherence to these dietary patterns among Iranian population from 2006 till 2017.

**Methods:**

We investigated on four examination waves of TLGS, including wave 1 (2006–2008), wave 2 (2009–2011), wave 3 (2012–2014), and wave 4 (2015–2017), using a validated and reliable food frequency questionnaire. Generalized Estimating Equations was used to assess secular trends in anthropometric, biochemical, and dietary variables across the study period. To identify general structure and secular trend of dietary patterns during each waves, principle component analysis (PCA) and K-mean cluster analysis were used, respectively.

**Results:**

After adjusting for potential confounders including age, sex, body mass index, and total energy intake, the carbohydrate and protein intake gradually increased and the total fat intake decreased during study period (*P*-value< 0.001), although total energy intake remained stable. During the study period, participants consumed noticeably less refined grains, solid fat, dairy products, and simple sugars. Snack and dessert consumption increased and meat intakes showed no significant changes during a decade (all *P*-values< 0.001). Three dietary patterns extracted using PCA, included: *Healthy dietary pattern* characterized by higher intakes of vegetable, fruit, dairy products, liquid oil, nuts and seeds, and honey and jam; *Western dietary pattern* featured by refined grain, solid fat, meat, snack and dessert, potato, and soft drink, and the *Mixed dietary pattern*, highlighted by tea and coffee, and simple sugar. Based on cluster analysis, 27.8% of participants in wave 4 followed a Western dietary pattern, and 34.1% followed the Mixed dietary pattern. The Healthy dietary pattern was stable among the study population during the last decade.

**Conclusions:**

The structure and the type of foods that participants preferred to eat changed since 2006, a new secular trend in dietary patterns, including a stability of *Healthy* dietary pattern, a decline of the *Western* dietary pattern and an increase in the *Mixed* dietary pattern was obsereved in our investigation.

## Introduction

Nutrition transition in the Middle East and North Africa (MENA) region, has been a result of the rapid demographic changes, social development, and urbanization [1, 2], demonstrated as a set of gradual dietary changes from healthy diets to a pattern of westernized foods [3]. According to the Global Burden of Disease (GBD) report, although the mean daily intake of some healthy foods including fruit, vegetables, fiber, and legume increased in the MENA region from 1990 to 2017, the burden of chronic diseases such as hypertension, type 2 diabetes, and cardiovascular disease rose, simultaneously [4–6], a finding indicating that concentrating on a single food per se cannot explain the link between fast rising trend in non-communicable diseases in the current decade and higher intakes of some healthy foods.

People eat meals consisting of foods in different combinations. As there are several synergistic and adversary interactions between nutrients, studying dietary patterns is a better approach to identify a holistic view of eating behaviors of populations rather than focusing on the single dietary factor [7, 8]. Principal component analysis (PCA) is one of the most commonly used approach to derive dietary patterns, which is based on data reduction methods and provide a more complete picture of diets for the study population.

Changes in dietary patterns among population have been a subject of growing interest in various regions [9–12]. Despite the rapid economic changes in China, dietary patterns of the Chinese population remained relatively stable from 1991 to 2009 [9]. Lim et al. demonstrated that approximately 40% of participants still follow a traditional Korean diet which remained relatively stable since 1998 to 2010; however, the secular trend of Western dietary patterns decreased from 30% in 1998 to 10% in 2010 [10].

As dietary intake is one of the main contributors to chronic diseases, a robust, quantitative understanding of dietary patterns is imperative for designing strategies to reduce national and global diet-related disorders [13]. Based on the important impact of globalization and urbanization on dietary pattern alterations as well as increased incidence of chronic diseases in the MENA, this study has two main objectives; first, to investigate the general structure and secular trend of dietary patterns extracted from the Tehran Lipid and Glucose Study (TLGS) and second, adherence to these dietary patterns among Iranian population from 2006 till 2017.

## Methods

### Study population

This study was conducted within the framework of the TLGS, a long term prospective population study initiated in 1998 to determine the prevalence of non-communicable disease risk factors and its outcomes among the urban Tehranian population; detailed have been previously described [14]. Briefly, the first examination was initiated in 1999 among 15,005 people, aged ≥3 years from district 13 of Tehran using the multistage cluster random sampling method. Participants undergoing a follow-up visit every 3 years, and data on any changes in demographic, anthropometric, reproductive and metabolic features and laboratory assessments were collected. The baseline examination was a cross-sectional study conducted from 1999 to 2001, and wave II (2002–2005), III (2006–2008), IV (2009–2011), V (2012–2014), and VI (2015–2017) were prospective follow-up waves. From the third examination wave of the TLGS (2006–2008), dietary assessment was begun on 3462 participants, who were randomly selected from 12,523 examined participants.

For the present study we investigated on four examination waves of TLGS, including waves 1 (2006–2008), 2 (2009–2011), 3 (2012–2014), and 4 (2015–2017). In each examination wave, we included all adults aged ≥18 years with complete dietary data for at least two waves. We excluded those who had only specific dietary changes, such as diet therapy for hypertension, dyslipidemia, and hyperglycemia (3372 participants excluded), furthermore, we also excluded 202 individuals because of under-or over-reporting of energy intakes (±3 standardized deviation). Finally, 2215 participants from wave 1, 1242 from wave 2, 1833 from wave 3, and 1218 from wave 4 were selected.

The study was approved by the research ethics committee of the Research Institute for Endocrine Sciences (RIES), Shahid Beheshti University of Medical Sciences, and written informed consent was obtained from the participants.

### Dietary assessment and food grouping

In each wave to assess the regular dietary intakes of participants over the previous year, during face-to-face interviews, trained dieticians gathered dietary data using a validated and reliable food frequency questionnaire (FFQ), for each food item on the FFQ, a portion size was specified using US Department of Agriculture (USDA) serving sizes whenever possible; if this was not possible, household measures were chosen and were then converted to grams. Energy and nutrient contents of food items were obtained from USDA food composition tables (FCT) because Iranian FCTs are incomplete. The Iranian FCT was used for traditional food items that are not listed in the USDA FCT.

To identify dietary patterns, dietary data were categorized into 17 groups, based on food and nutrient composition similarity [15] as follows: (1) Whole grains; (2) Refined grains; (3) Potatoes; (4) Dairy products; (5) Vegetables; (6) Fruits; (7) Legumes; (8) Meats; (9) Nuts and seeds; (10) Solid fats; (11) Liquid oils; (12) Tea and coffee; (13) Salty snacks; (14) Simple sugars; (15) Honey and jams; (16) Soft drinks; and (17) Snacks and desserts.

### Measurements

Height was measured by well-trained examiners which participants not wearing no shoes and was recorded to the nearest 0.5 cm. Weight was measured using digital scales (Seca, Hamburg, Germany) and was recorded to the nearest 100 g, while the subjects were minimally clothed and without shoes. Body mass index (BMI) was calculated as the weight divided by the square of the height (kg/m2). Waist circumference (WC) was measured at the level of the umbilicus to the nearest 0.5 cm, using a measuring tape in the standing position.

### Statistical analysis

All statistical analyses were performed using SPSS (version 16.0), with *P*-values < 0.05 considered significant. Normality of the distribution of variables was assessed by the Kolmogorov-Smirnov test and checked by Histogram. As plasma TG was skewed, the log transformation was used. Characteristics of participants were expressed as mean ± standard error (SE) for continuous variables and percentages for categorical variables. Generalized Estimating Equations (GEE) were used to assess secular trends in anthropometric, biochemical, and dietary variables across the years 2006 to 2017. Since the present study examined participants for a decade, all anthropometric measurements were adjusted for age, and nutrient intakes and dietary food groups were further adjusted for sex, BMI, and energy intakes of participants. Specifically, using GEE, anthropometric and nutritional variables were placed in dependent variables, and all the confounders were placed in the predictor’s part (categorical variables in the fixed and continuous variables in covariate parts). Finally, the adjusted mean ± SE of each variables were used as variable value. To identify dietary patterns during each separated examination wave, PCA was used, based on eigenvalues > 1, scree plot and factor interpretability. Variables with factor loadings of ≥0.3 were used in interpreting the factors. To assist interpretation, factors were rotated with the varimax procedure. Each dietary pattern was labeled by a descriptive name after the most important loading variables as *“Western dietary pattern*”, “*Healthy dietary pattern*” and “*Mixed dietary pattern*”. Finally, K-mean cluster analysis was used to assess the secular trend and adherence of dietary patterns among study population. In this method, three dietary patterns which were extracted from the PCA were added to the cluster analysis. Then, each participant give a specific score between 1 and 3 (based on the number of definite dietary patterns). Therefore, the percent of study population for each dietary pattern adherence can be calculated.

## Results

### Trends in anthropometric and biochemical parameters

Secular trends observed in anthropometric measurements during the four waves are shown in Table 1. In the age-adjusted model, there was an increase in BMI and waist circumference from 26.5 kg/m^2^ in wave 1 to 27.7 kg/m^2^ in wave 4 and from 88.2 cm in wave 1 to 93.6 cm in wave 4, respectively.

**Table 1:** General characteristics of the study population throughout the four waves

	Wave1 (2006–2008)	Wave2 (2009–2011)	Wave3 (2012–2014)	Wave4 (2015–2017)	P for trend
Participants (n)	2215	1242	1833	1218	
Age (year)	38.0 ± 0.2	39.6 ± 0.3	42.7 ± 0.3	46.2 ± 0.3	< 0.001
Female (%)	52.7	54.0	52.4	51.1	0.001
Waist (cm)	88.2 ± 0.2	91.8 ± 0.3	92.5 ± 0.3	93.6 ± 0.3	< 0.001
Body mass index (kg/m^2^)	26.5 ± 0.1	27.0 ± 0.1	27.4 ± 0.1	27.7 ± 0.1	< 0.001

Values are expressed as age-adjusted mean ± SE for continuous and percent for categorical variables. General estimate equation was used

### Changes in nutrient and energy intake

Table 2 shows the stability in total energy intakes among study population over the last decade. Moreover, percentage of energy from carbohydrate and protein intakes gradually increased (*P*-value< 0.001), whereas dietary total fat intake, saturated fatty acid, MUFA, and PUFA decreased slightly since the first wave (all P-value< 0.001). Interestingly, dietary sodium intake decreased from 2006 mg/1000Kcal in wave 1 to 1556 mg/1000Kcal in wave 4 (P-value< 0.001). Participants in wave 1 consumed 16.2 g/1000 kcal of dietary fiber which increased to 19.5 g/1000 kcal in wave 4; lastly, total sugar intake was remained stable throughout the study period.

**Table 2:** Changes in nutrient and energy intake among study population over the four waves

	Wave1 (2006–2008)	Wave2 (2009–2011)	Wave3 (2012–2014)	Wave4 (2015–2017)	P for trend
Energy (Kcal)	2383 ± 19.8	2518 ± 27.3	2396 ± 24.6	2282 ± 30.3	0.370
Carbohydrate (% of energy)	57.2 ± 0.1	58.7 ± 0.2	58.9 ± 0.1	59.7 ± 0.1	< 0.001
Protein (% of energy)	13.6 ± 0.0	14.8 ± 0.0	14.6 ± 0.0	15.1 ± 0.1	< 0.001
Fat (% of energy)	31.6 ± 0.1	29.8 ± 0.1	29.6 ± 0.1	29.1 ± 0.1	< 0.001
Saturated fatty acid (% of energy)	10.6 ± 0.1	9.8 ± 0.0	9.5 ± 0.0	9.3 ± 0.0	< 0.001
Monounsaturated fatty acid (% of energy)	11.0 ± 0.0	9.9 ± 0.0	9.8 ± 0.0	9.8 ± 0.0	< 0.001
Polyunsaturated fatty acid (% of energy)	6.6 ± 0.0	5.9 ± 0.0	5.9 ± 0.0	5.8 ± 0.0	< 0.001
Sodium (mg/1000Kcal)	2006 ± 31.9	1529 ± 12.3	1537 ± 11.4	1556 ± 12.2	< 0.001
Dietary fiber (gr/1000Kcal)	16.2 ± 0.1	19.4 ± 0.2	18.3 ± 0.1	19.5 ± 0.2	< 0.001
Total sugar (gr/1000Kcal)	52.6 ± 0.3	53.0 ± 0.3	54.0 ± 0.3	55.0 ± 0.3	0.166

Values are expressed as adjusted mean ± SE. All variables were adjusted by age, sex, body mass index and energy intake. General estimate equation was used

### Changes in dietary food groups

**Fig. 1:**
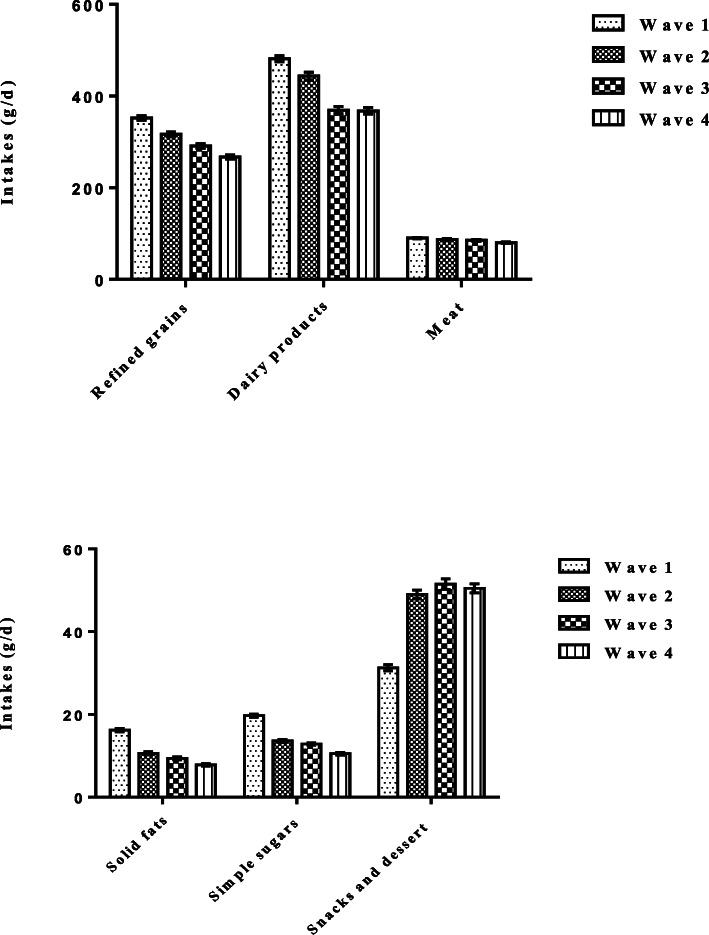
Food groups intake among study population from 2006 to 2017. Values are expressed as adjusted mean ± SE. All variables have been adjusted for age, sex, body mass index and energy intake. General estimate equation was used

Figure 1 indicates food groups consumed among the population through the four waves. During the study period, participants consumed notably refined grains, solid fat, and simple sugar (all *P*-values< 0.001). In addition, intakes of dairy products decreased significantly during the last decade (P-value< 0.001). During the study period, meat consumption had no significant changes among study participants. Interestingly, snack and dessert consumption increased rapidly, especially in the third wave (all P-values < 0.001). Moreover, fruit and vegetable intake remained stable throughout the study period (data not shown).

### Changes in dietary patterns

Table 3 shows the factor loading of food items throughout four waves. The first dietary pattern explored by PCA was the *Healthy dietary pattern* which was characterized by higher intakes of vegetable, fruit, dairy products, liquid oil, nuts and seeds, and honey and jam and this factor explained 12.0% of the variance; the second dietary pattern was the *Western dietary pattern* which was featured by refined grain, solid fat, meat, snack and dessert, potato, and soft drink and lastly and This factor explained 9.7% of the variance, the *Mixed dietary pattern* extracted was highlighted by tea and coffee, and simple sugar, explaining 8.2% of the variance. In the second wave, the 3 dietary patterns were changed as follow: first of all, meat, soft drink, and potato were added to the *Healthy* dietary pattern; secondly, people ate more legumes along with their *Western dietary pattern*; and finally, solid fat was concurrently expressed from *Western* to the *Mixed* dietary pattern, in comparison to the previous wave, participants in wave 3 modified their dietary patterns. Honey and jam intakes was not loaded in the *Healthy* dietary pattern, and potato intake shifted from the *Healthy* to the *Western* dietary pattern; the fourth wave, soft drink, meat, and potato intake were loaded in the *Western* dietary pattern. Interestingly, dietary refined grain has been shifted slightly from the *Western* to the *Mixed* dietary pattern. (The communalities of food groups in all the waves were shown in the supplementary Table 1).

**Table 3:** Food groups loadings for 3 dietary patterns found by principle component analyses among study population

Food groups	Wave1			Wave2			Wave3			Wave4		
	H	W	M	H	W	M	H	W	M	H	W	M
Vegetables	0.591	–	–	0.631	–	–	0.513	–	–	0.693	–	–
Fruits	0.683	–	–	0.685	–	–	0.621	–	–	0.682	–	–
Dairy products	0.544	–	–	0.511	–	–	0.513	–	–	0.418	–	–
Liquid oils	0.534	–	–	0.478	–	–	0.653	–	–	0.334	–	–
Nuts and seeds	0.522	–	–	–	–	–	0.319	–	–	0.422	0.314	–
Honey and jams	0.366	–	–	0.434	–	0.324	–	–	–	0.319	–	0.379
Soft drinks	–	0.446	–	0.487	–	–	0.489	–	0.405	–	0.585	–
Snacks and desserts	–	0.447	–	–	0.721	–	–	0.708	–	–	0.701	–
Meats	–	0.459	–	0.407	–	–	0.460	–	–	–	0.383	–
Refined grains	–	0.602	–	–	0.471	–	–	–	0.423	–	–	0.474
Potatoes	–	0.446	–	0.447	–	–	–	0.372	–	–	0.503	–
Solid fats	–	0.536	–	–	–	0.337	–	–	0.470	–	–	0.301
Salty snacks	–	0.309	–	0.312	–	–	–	–	–	0.519	–	–
Simple sugars	–	–	0.711	–	–	0.830	–	–	0.764	–	–	0.735
Tea and coffee	–	–	0.765	–	–	0.747	–	–	0.586	–	–	0.574
Whole grains	–	–	–	–	0.337	–	–	–	–	–	–	–
Legumes	–	–	–	–	0.726	–	–	0.785	–	–	0.639	–
Cumulative variance %	12.0	21.7	30.0	13.3	24.2	33.6	12.7	22.5	31.8	12.1	23.6	33.0

Values less than 0.3 were excluded for simplicity

*H* Healthy dietary pattern, *W* Western dietary pattern, *M* Mixed dietary pattern

### Adherence of study population to each dietary pattern

**Fig. 2:**
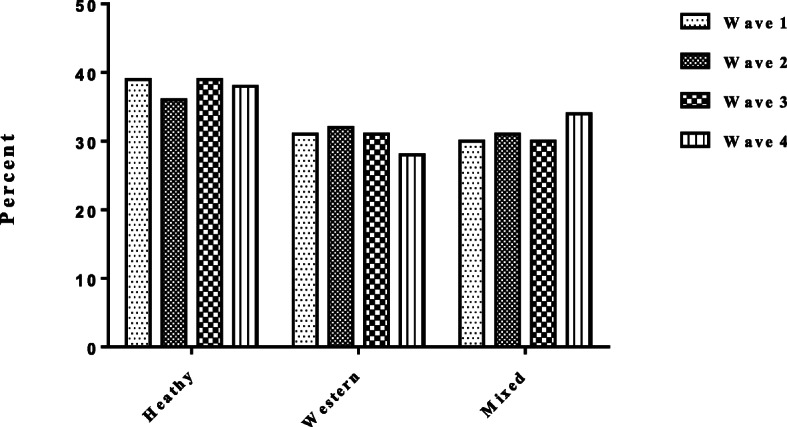
Adherence to 3 dietary patterns among study population in Iran from 2006 to 2017. K-mean cluster analysis was used

Based on cluster analysis, the secular trends in dietary patterns are presented in Fig. 2; overall the trend showed a decline in the *Western* dietary pattern from 31.2% in wave 1 to 27.8% in wave 4, and an increase in the *Mixed* dietary pattern from 29.7% in wave 1 to 34.1% in wave 4. Finally, the *Healthy* dietary pattern remained stable among the study population during the last decade.

## Discussion

In the present study which analyzed dietary data collected from four waves over the course of a decade (from 2006 to 2017), we derived three dietary patterns using factor analysis mostly centered on these food groups: 1. A *Healthy* dietary pattern, characterized by vegetable, fruit, dairy products, liquid oil, and nuts and seeds; 2. A *Western* dietary pattern featured by soft drinks, snack and dessert, meat, refined grain, and solid fat intake, and 3. A *Mixed* dietary pattern highlighted by tea and coffee, and simple sugars. Our findings indicated that the structure of these dietary patterns did not seem to be stable over a decade, indicating that the type of food groups that population chose to eat in combination had changed since 2006. Results demonstrating a secular trend in dietary patterns including an emerging adherence of study population to the *Mixed* dietary pattern, maintenance of the *Healthy* dietary pattern, and a decline in the *Western* dietary pattern.

Most previous epidemiological studies which focused on dietary patterns, have analyzed the relation of dietary pattern with risk of chronic diseases [16–21], and only a few of them have investigated the secular trend of dietary patterns over time [9, 10, 22, 23]; mostly three common dietary patterns have been identified throughout these investigations. The Healthy or Prudent dietary pattern which is mostly based on fruit, vegetables, dairy products, and liquid oil, Unhealthy or Westernized dietary pattern, mostly characterized by solid fat, snack, soda, and meat; however, the third one, mostly named as modified, new or mixed dietary pattern, differ in each study with different factor loading and food items. Results of the *Healthy* and *Western* dietary patterns loaded in the current study are mostly similar to other studies [9, 21]. Adherence of our study population to the three dietary patterns is similar to those of a Korean population, in which the number of participants following the Western dietary pattern declined, and the new dietary pattern increased in population over time [10].

According to the results of this study, energy intakes of study population remained stable since the first wave**.** However, the percent of energy from carbohydrate and protein intakes increased whereas percent of energy from fat and all its subtypes (saturated, mono- and polyunsaturated fatty acids) decreased. People have become increasingly aware of the health benefits of vegetable oils and it seems that the sources of fat intake have changed during the last decade, with a significant shift from intakes of solid fat to the liquid oil [24]. Likewise, sources of protein intake have changed from animal to plant based, including legumes.

In term of the trend in food consumption, our findings indicate that fruit and vegetable intakes remained consistent, indicating that policy based-approaches must be considered to increase fruit and vegetable intakes, as consumption of these food groups is barely possible for populations with low income. Whole grain, increased significantly since the first wave, which was in contrast with the trend of other MENA region countries [4]. In the current study, intake of meat as a protein source was stable over the last decade, a finding contrary to the results of other Asian countries, including China and India, where meat intake had increased since westernization [25]. It is important to note that with the growing rate of urbanization, study population consume more snacks and desserts. One of the important points of the current study is that dietary dairy intakes decreased significantly throughout the waves; this finding has been confirmed by the World Health Organization STEP wise approach to Surveillance (STEPS), which indicated that only approximately 18% of Iranian population meet the appropriate amount of dairy intakes [26].

Based on the results of the current study, the percent of study population who follow the *Mixed* dietary pattern has increased since the wave 1; this dietary pattern includes of simple sugar, tea and coffee, and whole grain, and is very similar to the traditional dietary pattern of Iranian population. Simple sugar and tea and coffee were loaded in all four waves, showing these food items to be deeply rooted in the traditional dietary pattern of Iranian culture. Interestingly, in the second and mostly the third wave unhealthy food items such as refined grain, and solid fat were added to the *Mixed* dietary pattern, possibly influence by modified Iranian meals like a western style dinner. The traditional Iranian diet is wheat-based. Tea is the major beverage and dairy products such as yoghurt and cheese was consumed widely [2]; however, the consumption of dairy products decreased since the previous decade. It is noteworthy that about 38% of our study populations try to maintain a healthy dietary pattern; however, intakes of food groups have changed and they accepted Western-style foods are more consumed, based on changing environmental factors, indicating that Iranians have modified their dietary pattern; in other words it is not totally westernized, although many of unhealthy foods have been added to the traditional dietary patterns of our study population.

The strength of the present study is as follow. Firstly, it was the first study investigating the secular trend of Iranian population dietary patterns and the adherence to these dietary patterns during the last decade. Secondly, the longitudinal design of the study, by which we could track changes in dietary patterns individually. Besides, we believe that our comparisons between years were not affected by the cohort effect, not only because all the subjects were included in at least two waves but also because we adjusted all the results by age in the first wave.

One of the limitations of the present study is that some food groups including dairy products and meat intakes were not categorized into subgroups. For example, dairy products include of low fat dairy, high fat dairy, or meats include of egg, red meat, processed meat, poultry, and fish. Therefore, it is not clear that higher factor loading of these food groups in one of the three dietary patterns is because of which sources of food items; however, we consider all the foods which were categorized in a same groups of the food pyramid. Another important limitation is that the number of foods available in the food supply exceeds by far the number of those available in food composition tables so the present study was unable to capture all the changes in dietary intake, particularly of packaged processed foods. Recall bias was also an inevitable problem when asking participants to remember and report dietary intakes. Finally, information about diet therapies for obesity had not been gathered by demographic questionnaire and was not in the scope of TLGS. Because of that we could not exclude participants with diet therapy for obesity.

## Conclusions

Overall, it is clear that the structure and the type of foods that this population preferred to had changed since 2006. We report here in a new secular trend in dietary patterns, including a stability of the Healthy dietary pattern, a decline of the Western dietary pattern and an increase in the Mixed dietary pattern. These findings may recommend community policies on successful points, areas for greater attention, and opportunities to improve the dietary habits of the population living in Iran.

## Supplementary information

**Additional file 1 Supplementary Table 1.** Communalities of dietary food groups in each wave of TLGS.

## Data Availability

All data generated or analyzed during this study are included in this published article.

## Abbreviations

BMI: Body mass index
BP: Blood pressure
DBP: Diastolic blood pressure
FBS: Fasting blood glucose
FCT: Food composition tables
FFQ: Food frequency questionnaire
GBD: Global Burden of Disease
GEE: Generalized Estimating Equations
MENA: Middle East and North Africa
PCA: Principle component analysis
SE: Standard error
STEPS: STEP wise approach to Surveillance
TC: Total cholesterol
TLGS: Tehran Lipid and Glucose Study
USDA: US Department of Agriculture
WC: Waist circumference
