# Supplementary material for: Secular trend in dietary patterns of Iranian adults from 2006 to 2017: Tehran lipid and glucose study
Source: Nutr J. 2020 Oct 3;19:110. doi: 10.1186/s12937-020-00624-x (PMC7533031; doi:10.1186/s12937-020-00624-x)
Supplement: Supplementary file 1 — Additional file 1 Supplementary Table 1. Communalities of dietary food groups in each wave of TLGS. [file 12937_2020_624_MOESM1_ESM.docx]

| Supplementary table 1. Communalities of dietary food groups in each wave of TLGS. | | | | |
| --- | --- | --- | --- | --- |
|  | Wave 1 | Wave 2 | Wave 3 | Wave 4 |
|  | Communality | Communality | Communality | Communality |
| Whole grains | 0.11 | 0.13 | 0.11 | 0.04 |
| Refined grains | 0.38 | 0.29 | 0.23 | 0.31 |
| Potatoes | 0.26 | 0.24 | 0.19 | 0.26 |
| Dairy products | 0.31 | 0.26 | 0.28 | 0.26 |
| Vegetables | 0.39 | 0.42 | 0.34 | 0.49 |
| Fruits | 0.48 | 0.48 | 0.40 | 0.47 |
| Legumes | 0.06 | 0.60 | 0.63 | 0.48 |
| Meats | 0.28 | 0.22 | 0.25 | 0.28 |
| Nuts and seeds | 0.28 | 0.07 | 0.11 | 0.30 |
| Solid fat | 0.29 | 0.12 | 0.29 | 0.10 |
| Liquid oil | 0.29 | 0.24 | 0.43 | 0.20 |
| Tea and coffee | 0.58 | 0.56 | 0.44 | 0.34 |
| Salty snacks | 0.09 | 0.15 | 0.14 | 0.27 |
| Simple sugars | 0.53 | 0.70 | 0.58 | 0.57 |
| Honey and jam | 0.16 | 0.29 | 0.06 | 0.25 |
| Soft drinks | 0.27 | 0.27 | 0.28 | 0.38 |
| Snacks and desserts | 0.25 | 0.61 | 0.58 | 0.53 |

Extraction method: Principle Component Analysis (PCA)
